# Machine-driven vs. manual nickel-titanium instrumentation of canine teeth in dogs: a high resolution micro-CT evaluation of canal geometry and obturation quality

**DOI:** 10.3389/fvets.2026.1960227

**Published:** 2026-09-14

**Authors:** Johanna Wunderl, Matthias Eberspächer-Schweda, Stephan Handschuh, Alexander Tichy, Eva Schnabl-Feichter

**Affiliations:** 1 Vetklinikum GmbH & Co KG, Department for Dentistry, Vienna, Austria; 2 VetCore Facility for Research/Imaging Unit, University of Veterinary Medicine Vienna, Vienna, Austria; 3 Bioinformatics and Biostatistics Platform, Department of Biomedical Sciences, University of Veterinary Medicine Vienna, Vienna, Austria; 4 Small Animal Surgery, Department for Small Animals and Horses, University of Veterinary Medicine Vienna, Vienna, Austria

**Keywords:** canine tooth, dogs, endodontic treatment, micro-CT, Ni-Ti, root canal treatment

## Abstract

Root canal treatment in canine teeth of dogs represents a technically demanding procedure due to complex root canal anatomy and large canal dimensions. The present study aimed to compare different endodontic instrumentation techniques and to evaluate their effects on canal geometry, obturation quality, and procedural safety using high-resolution micro-computed tomography (micro-CT). In addition, the diagnostic value of cone-beam computed tomography (CBCT) in the assessment of postoperative findings was investigated. Thirty extracted canine teeth were randomly assigned to three groups (*n* = 10) and instrumented using two different machine-driven nickel-titanium (Ni-Ti) systems or manual Ni-Ti hand files. Pre- and post-treatment micro-CT scans were used for quantitative analysis of canal morphology, post-to-pre canal volume ratio, air inclusion, and discrepancies between canal diameter and instrument size. CBCT imaging was performed for post-treatment evaluation. Mixed-model analysis revealed a significant overall effect of instrumentation group on the discrepancy between canal diameter and instrument size (*p* = 0.008). Pairwise comparisons demonstrated significantly greater discrepancies for manual instrumentation than for one of the machine-driven systems, whereas the remaining comparisons were not statistically significant after Sidak correction. No significant differences were observed in the post-to-pre canal volume ratio between groups (*p* = 0.464). A significant group effect was identified for internal air volume (*p* = 0.017), with manual instrumentation being associated with greater internal air volume, indicating less homogeneous obturation. Procedural complications occurred in 26.7% of specimens and were more frequent for the less experienced operator. CBCT imaging demonstrated radiolucent zones at the dentin–filling interface that were not confirmed as voids or debris by micro-CT and were therefore attributed to imaging artifacts. Machine-driven Ni-Ti systems provided more reproducible canal preparation and were associated with more homogeneous obturation despite achieving a comparable post-to-pre canal volume ratio. Moreover, high-resolution micro-CT demonstrated that radiolucent CBCT findings at the dentin–filling interface predominantly reflected imaging-related artifacts rather than true treatment deficiencies, highlighting the importance of cautious interpretation of postoperative CBCT examinations in veterinary endodontics.

## Introduction

Tooth-preserving procedures such as root canal treatment are no longer exclusively relevant to human dentistry but have gained increasing importance in veterinary medicine over recent decades (1–3). Advances in materials, instrumentation, and imaging modalities have expanded the indications for endodontic treatment in companion animals, particularly in dogs.

Root canal treatment is considered one of the most technically demanding procedures in dentistry, requiring the coordinated use of multiple instruments, chemical irrigants, and carefully sequenced clinical steps. These steps typically include the preparation of an access cavity, removal of diseased pulp tissue, mechanical and chemical shaping of the root canal system, obturation with appropriate filling materials to eliminate potential niches and pathways for bacterial colonization, and definitive coronal restoration to prevent microleakage (4, 5).

Endodontic treatment in dogs and cats differs substantially from human endodontics, primarily due to species-specific variations in tooth size, root length, canal diameter, and pulp chamber morphology (1, 2). These anatomical differences can complicate adequate debridement and obturation, particularly in large canine teeth.

Root canal treatment may be performed in both single- and multi-rooted teeth. In veterinary dentistry, strategically and functionally important teeth, such as the maxillary and mandibular canine teeth as well as the carnassial teeth (maxillary fourth premolar and mandibular first molar) are frequently treated endodontically due to their essential role in prehension, mastication, and working performance (1, 6).

Preservation of these teeth is especially important in working and service dogs, including police, military, hunting, and therapy dogs, where tooth loss may significantly impair function and quality of life (2).

Accurate intraoperative and postoperative imaging is essential for both the success and follow-up evaluation of root canal treatment. Conventional dental radiography remains the most commonly used imaging modality in veterinary practice, as it is readily available, cost-effective, and allows intraoperative use without repositioning the patient. However, its two-dimensional nature limits the assessment of complex root canal morphology.

Cone-beam computed tomography (CBCT) has become an established imaging tool in dentistry, offering high-resolution three-dimensional visualization of teeth and root canal systems and has demonstrated significant diagnostic advantages in both clinical and research settings (7, 8). In human endodontics, CBCT has proven particularly valuable for evaluating canal anatomy, treatment outcomes, and procedural complications (7).

The primary aim of the present study was to compare two mechanically driven reciprocating Ni-Ti instrumentation techniques with manual Ni-Ti instrumentation in canine teeth of dogs and to quantitatively evaluate their effects on root canal geometry and obturation quality using high-resolution micro-CT. A secondary aim was to qualitatively compare radiolucent findings observed at the dentin–filling material interface on postoperative CBCT with the corresponding high-resolution micro-CT findings.

We hypothesized that the three instrumentation techniques would differ in their effects on root canal geometry and obturation quality. In addition, we hypothesized that radiolucent interface zones observed on postoperative CBCT would not necessarily correspond to true structural defects, such as voids or residual debris, when evaluated using high-resolution micro-CT.

## Materials and methods

A total of 30 lower and upper dog canines, which were extracted for reasons unrelated to this study, were analyzed. Teeth were fixed in neutral buffered formalin and subsequently stored in 70% ethanol. Prior to treatment, a micro-CT scan of each tooth had been acquired as an anatomical reference as part of a preceding study. The sample size of the present investigation was therefore constrained by the availability of canine teeth with corresponding pre-treatment micro-CT datasets that met the predefined inclusion criteria. Consequently, an *a priori* power analysis was not performed.

The samples were categorized according to weight, age, and jaw position (upper/lower) following the modified Triadan numbering system (104, 204, 304, 404) and randomly assigned to three groups (9). The 30 canine teeth were divided into three groups of 10, each assigned to one of the following preparation techniques. Within each group, five teeth were instrumented by a pre doctoral specialist (operator no. 1) and five by a board-certified veterinary dentist DAVDC/DEVDC (operator no. 2).

- Group A: Control Cut Rotary Flex Ni-Ti files by iM3^a^ (machine-operated with the iM3 C Flex endomotor^b^).
- Group B: Vet Flex and Preserve Ni-Ti files by Dentsply^c^ (machine-operated with the iM3 C Flex endomotor^b^).
- Group C: Hand files: Ni-Ti files by iM3^d^ (K-Reamer; Figure 1).

**Figure 1:**
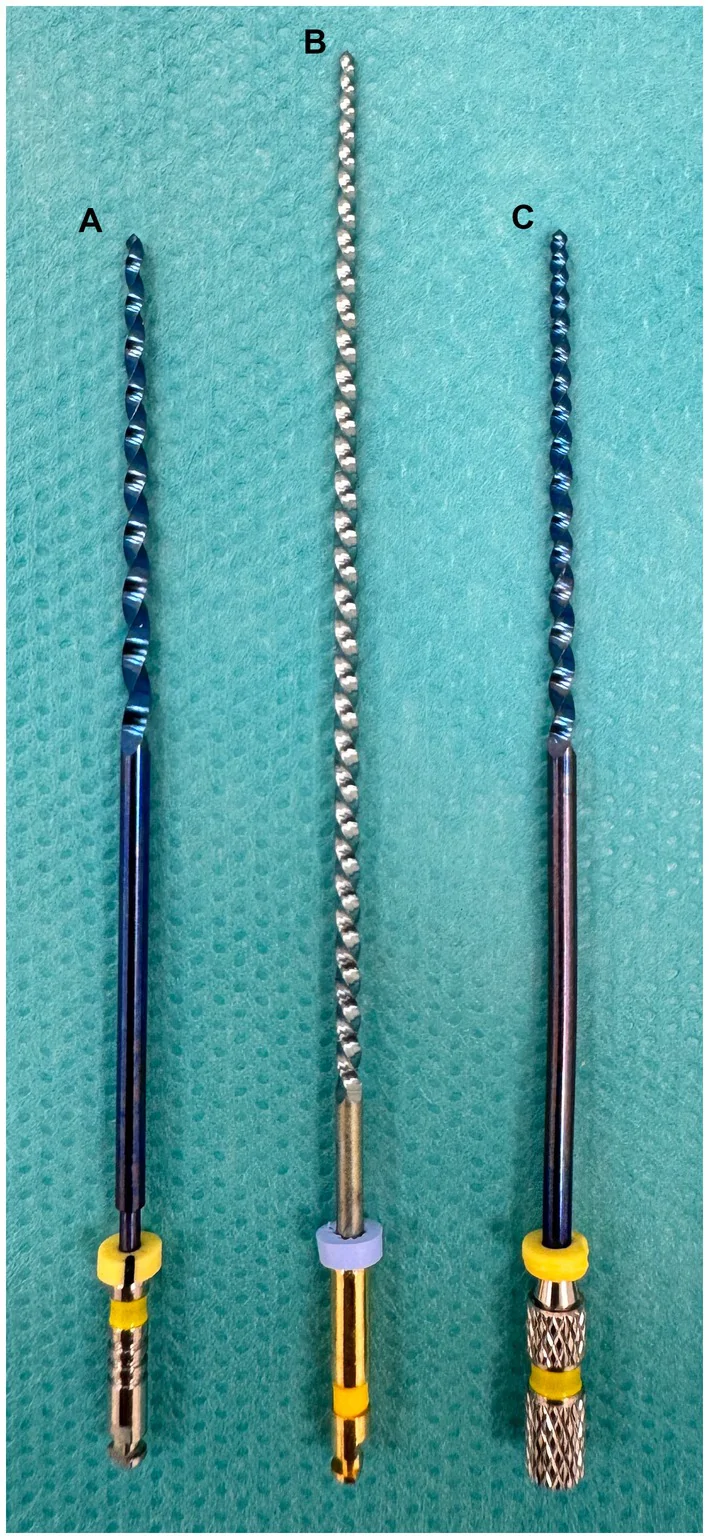
Endodontic instruments used in the three instrumentation groups (From left to right): **(A)** Group A: Control Cut Rotary Flex Ni-Ti file (iM3). **(B)** Group B: Vet Flex & Preserve Ni-Ti file (Dentsply Sirona). **(C)** Group C: Ni-Ti hand K-reamer (iM3).

To standardize instrument wear while reflecting routine clinical practice, each instrument was used for the preparation of a maximum of two teeth before being replaced with a new instrument set. Prior to treatment, each tooth was individually embedded in a polycarbonate block^e^ (2, 6) and subjected to standardized procedures (preparation, imaging, chemical preparation, filling, and restoration; Figure 2). The root canal treatment protocol followed the standard clinical procedure routinely applied at our clinic. A complete list of all materials and instruments used is provided in Supplementary Table S1.

**Figure 2:**
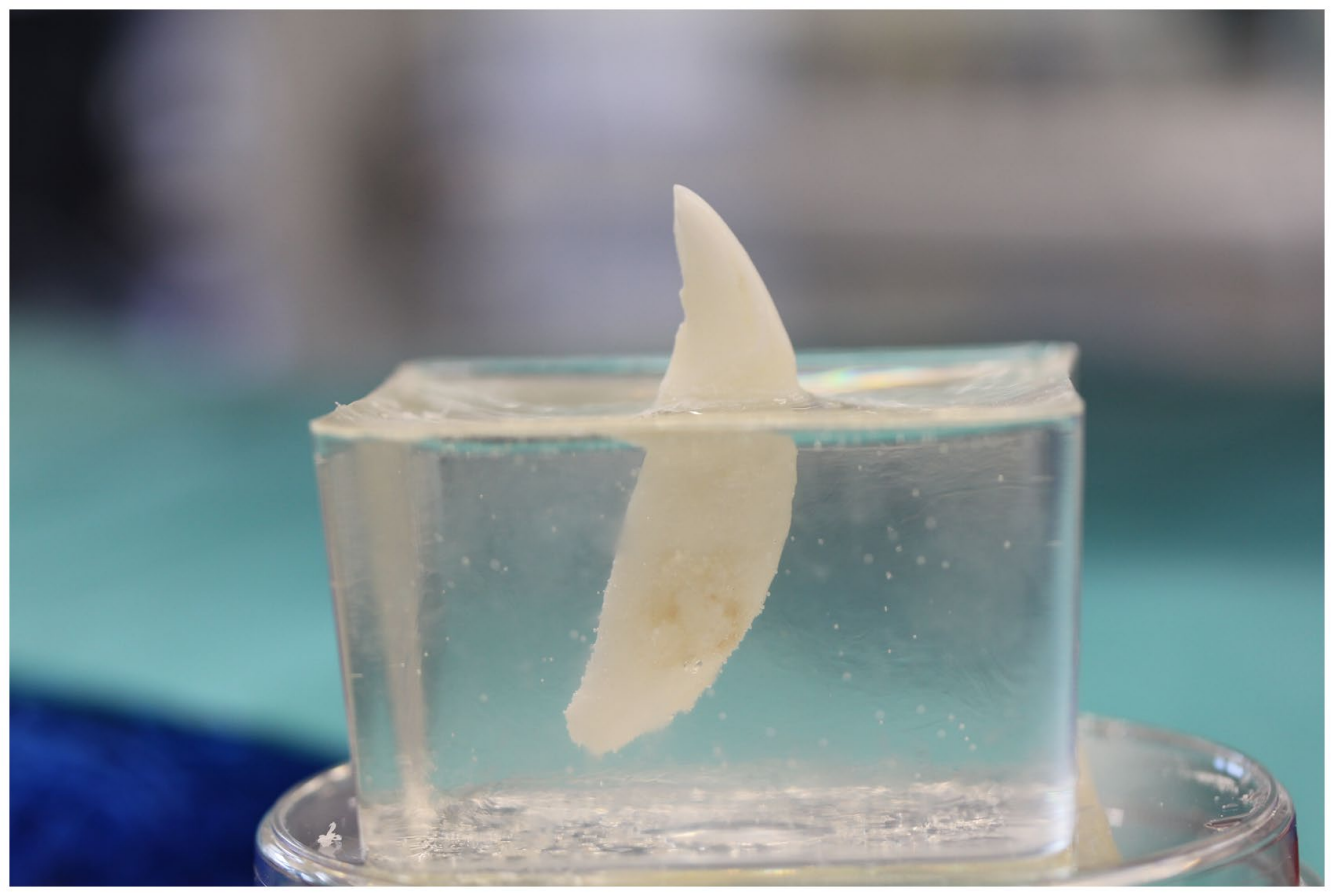
Representative canine tooth specimen individually embedded in a polycarbonate block prior to root canal preparation.

### Access

Each tooth was individually positioned in polycarbonate at the height of the cementoenamel junction (CEJ) and allowed to dry for at least 48 h. Enamel cleaning and disinfection were performed using cotton swabs soaked in 96% ethanol. To simulate a tooth fracture, 25% of the crown length was measured and horizontally sectioned using a rotating diamond disk under permanent water cooling. A round diamond bur #2^f^ one mm in diameter was subsequently used to prepare a coronal cavity for at least 6 mm in depth toward the “fracture site” in groups A and B.

For group C, in which nickel–titanium (Ni-Ti) hand files were used, an auxiliary access hole was created as described in many studies approximately 2 mm above the CEJ (1, 10). The coronal cavity was subsequently enlarged using Gates-Glidden (GG) burs (sizes 1-6)^g^ in combination with the lubricant Glyde^h^. The burs were introduced passively into the canal, and active dentin removal was performed exclusively during withdrawal.

### Preparation

Pulpal extirpation was performed with single-use standard nerve needles^i^ of different sizes. The first ISO coded endodontic file to reach the apex (FF = first file) was identified to determine the working length in millimeters, which was verified radiographically and with a silicone stopper measured on an endodontic ruler. The following endodontic files were selected according to their increasing ISO size and a silicone stopper was always positioned at the predetermined working length.

In group A, Control Cut Rotary Flex Ni–Ti files were operated using a standardized reciprocating motion at 300 rpm and 2.0 Ncm, with rotational angles of 150° clockwise and 30° counterclockwise. The larger forward angle was selected to correspond to the instrument’s right-handed cutting design, in which active cutting occurs during clockwise rotation. The settings were used as the recommended presetting from the iM3 C Flex endomotor (11).

In group B, Vet Flex & Preserve Ni-Ti files^c^ were operated under standardized reciprocating conditions at 300 rpm and 2.0 Ncm, using a 150° counterclockwise and 60° clockwise rotational movement. As the instrument design corresponds to a left-handed cutting configuration, the larger angular displacement (150°) was assigned to the counterclockwise phase. These parameters were selected according to the manufacturer’s recommendations (12, 13).

The specimens in group C were instrumented manually using Ni-Ti K-reamers^d^ with a clockwise ¼-turn–pull technique, also with a right-handed cutting design.

After each endodontic file, a standardized irrigation protocol for chemical preparation was applied which remained consistent across all three preparation techniques (14, 15). The irrigation procedure performed using a special side vented irrigation needle 27G^j^ consisted of the following steps: First, sodium hypochlorite^k^ 5.25% (NaOCl) was used to flush and irrigate the root canal, followed by recapitulation with an ISO 15^d^ (K-Reamer, Ni-Ti) file to agitate dentinal debris. The solutions were stored at 4 °C according to the manufacturer’s recommendations and brought to room temperature prior to use (16). Suction was then performed using an empty syringe together with an irrigation needle. Subsequently, sterile sodium chloride 0.9%^l^ (NaCl) was used for final irrigation, followed again by suction.

Glyde^h^ was used as a root canal conditioner with each endodontic file to enhance canal lubrication and reduce the risk of instrument fracture (17). The final file (LF = last file) was defined as the largest file that just reached the apex without altering the established working length. A uniform chemical irrigation protocol was applied across all three techniques. For the final irrigation, 17% EDTA^m^ was introduced into the root canal and left in place for 1 min before being removed with suction. This was followed by a one-minute retention of NaOCl, which was subsequently suctioned. Finally, the canal was irrigated with NaCl and dried using ISO-standardized paper points^n^.

### Procedural complications

Procedural complications occurring during root canal instrumentation were recorded for each tooth. For each incident, the instrument involved, the success or failure of fragment retrieval, and the operator performing the procedure were documented. Multiple complications occurring in the same tooth were recorded as separate incidents. If retrieval of a fractured instrument was unsuccessful, the affected tooth was excluded from further treatment and replaced by another tooth to maintain the predefined group size of 10 specimens. Procedural complications were summarized descriptively as absolute numbers and percentages, including their distribution according to instrument type and operator experience.

In addition to the systematic documentation of procedural complications, all instruments were visually inspected before and during use for macroscopic deformation and adherent surface debris. These observations were qualitative and were not subjected to standardized scoring or statistical comparison between groups.

### Obturation

The first step of the obturation procedure was the selection of an appropriate gutta-percha point^o^ corresponding to the ISO size of the final file. GuttaFlow bioseal^p^ was then applied using the designated application tip. A Lentulo spiral was used to distribute the GuttaFlow bioseal evenly throughout the root canal system (18).

For the single-cone technique, the prepared gutta-percha point^o^, previously disinfected for 1 min in 5.25% NaOCl (5) and flushed in 96% ethanol to remove the surface deposits that may form, was inserted into the canal. The gutta-percha point was then severed using a heated excavator. Material compaction was performed using pluggers^q^ for vertical compaction. The dentin wall of the coronal cavity was cleaned with different excavators^r^ and standardized intraoral radiographs were obtained as part of the routine clinical protocol and at the operator’s discretion (Figures 3A–E).

**Figure 3:**
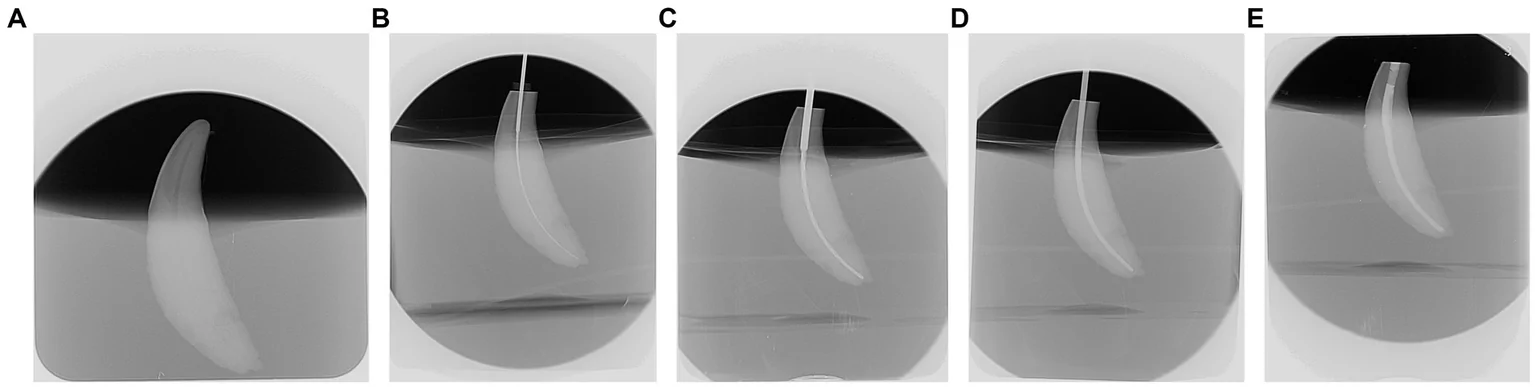
Representative radiographic sequence illustrating the different stages of root canal treatment. **(A)** Pre-treatment radiograph showing the initial canal anatomy; **(B)** working-length determination using the first endodontic file (first file); **(C)** radiograph showing the final endodontic file (last file); **(D)** master-cone radiograph prior to obturation; **(E)** post-treatment radiograph demonstrating the final root canal filling.

### Restoration

A so called “sandwich technique” was used for the final access restoration, involving the bonding of two different restorative materials. Glass ionomer cement^s^ was applied in a layer on top of the obturation not exceeding 2 mm in thickness, followed by light polymerization with blue light using 500–1,000 mW/cm^2^ and approximately 470 nm wavelength for 20 s at a distance of 1–1.5 mm from the tooth surface.

Adhese Universal Viva Pen^t^ (bonding agent) was then applied according to the manufacturer’s instructions and again light-cured with blue light for 20 s at a distance of 1–1.5 mm from the tooth surface. The final restoration was completed using Tetric EvoCeram^u^ (packable composite), which was also light-cured for 20 s.

The composite was polished using SHOFU^v^ polish to obtain a smooth surface matching the surrounding enamel and finally the surface was sealed with G-Coat Nanoseal^w^ under light-curing.

### CBCT

All treated teeth were scanned postoperatively using a CBCT (Planmed Verity®, Planmed Oy, Helsinki, Finland). The CBCT scans were acquired using the following parameters: scan time 18 s, reconstruction time approximately 30 s, voxel size 0.2 mm, tube voltage 84 kV, tube current 4 mA and exposure time 10.0 s. Image acquisition parameters were selected according to the manufacturer’s specifications and adapted to the experimental setup. Volumetric datasets were exported in DICOM format for subsequent analysis (19).

### Micro-CT

All scans were acquired using a micro-CT μCT35 system (SCANCO Medical AG, Brüttisellen, Switzerland).

Untreated teeth were mounted in Parafilm-sealed sample holders containing 70% ethanol. Scans of untreated teeth were acquired at 70 kVp / 114 μA and using a 0.5 mm Aluminum (Al) filter. Integration time was 800 ms (detector binning = 2) and the angular increment was 0.36°. Depending on the size of the tooth, isotropic voxel size in the datasets of untreated teeth was either 20 μm (large teeth, 20 mm sample holder, 20.5 mm field of view) or 12 μm (small teeth, 12 mm sample holder, 12.3 mm field of view).

Treated teeth surrounded by trimmed polycarbonate blocks were mounted in 20 mm sample holders in air. Scans of treated teeth were acquired at 70 kVp / 114 μA and using a 0.1 mm copper (Cu) filter. Integration time was 800 ms (detector binning = 2) and the angular increment was 0.36°. The isotropic voxel size in reconstructed datasets of treated teeth was 20 μm.

Reconstructed image volumes were exported in DICOM (*.dcm) file format.

### CBCT and micro-CT comparison

Post-treatment CBCT datasets were visually assessed for radiolucent areas at the dentin–filling material interface. During subsequent micro-CT image analysis, all specimens were visually inspected at the voxel level by a study operator with particular attention to whether radiolucent findings identified on CBCT corresponded to actual voids, air inclusions, debris accumulations, or structural discontinuities in the corresponding micro-CT datasets. The comparison between CBCT and micro-CT findings was performed qualitatively and was not subjected to statistical analysis.

### Image processing and analysis

#### Image registration and pre-treatment segmentation

DICOM image volumes were imported into the 3D analysis and visualization software AMIRA *(FEI Visualization Sciences Group, part of Thermo Fisher Scientific, Mérignac Cédex, France, version 2023.2*) and converted to Amira mesh (*.am) file format. Pre- and post-treatment image volumes were imported into a project template and spatially co-registered (rigid transformation) based on normalized mutual information using the *Register Images* tool.

For noise reduction, pre-treatment micro-CT datasets were filtered using a *Bilateral filter* (interpretation: 3D, kernel size 3*3*3, similarity = 10,000). The Amira *Segmentation Editor* was used to segment the mineralized tooth from the pulp using a fixed threshold (2600). The initial segmentation of the pulp was then further processed using a closing operation (3 pixel dilation followed by 3 pixel erosion) followed by smoothing using the command *Smooth Labels* (size = 3, mode = 3D volume). The volume of the tooth and pulp was measured using the *Material Statistics* tool.

#### Centerline generation and cross-sectional analysis

Next, a *B-Spline* (degree: 3, samples 201) was set for the entire length of the pulp by manually placing markers in the center of the pulp based on the tomograms (*Ortho Slice* module). The B-Spline was converted to a spatial graph (module *Line Set to Spatial Graph*) and smoothed using the module *Smooth Line Set* (smoothing: 0.5, iterations: 100). Finally, a Tcl script - originally published as supplement to Johnson Chacko et al. (20) and used here with slight modifications regarding the pixel size—was executed using the *Tcl Command Module*. This script used a *Trajectory* to move a *Slice* of the pulp segmentation (binary label field) along each point of the spatial graph of the pulp. The orientation of the slice was always perpendicular to the curvature at the respective position of the curve. The extracted cross-sectional images of the binary segmentation mask of the pulp (*Extract Image*) were exported as *.tif images and analyzed using a custom Fiji macro, which measured multiple parameters such as pulp area, perimeter, major and minor axis of ellipse fit, Feret’s diameter, Feret Angle, circularity, roundness, and aspect ratio. The custom Fiji macro automatically processed all 201 cross-sectional images of each specimen. Although multiple geometric parameters were recorded, Feret’s diameter was used as the measure of canal diameter for the file-to-canal diameter analysis reported in the present study.

As each spatial graph curve included exactly 201 points (as initially defined in the *B-Spline*), each step from one cross-sectional image of the pulp to the next represented exactly 0.5% of the pulp length. This allowed to compare relative changes in pulp area and shape across different teeth, despite the different size of measured teeth (20) (Figures 4A–E).

**Figure 4:**
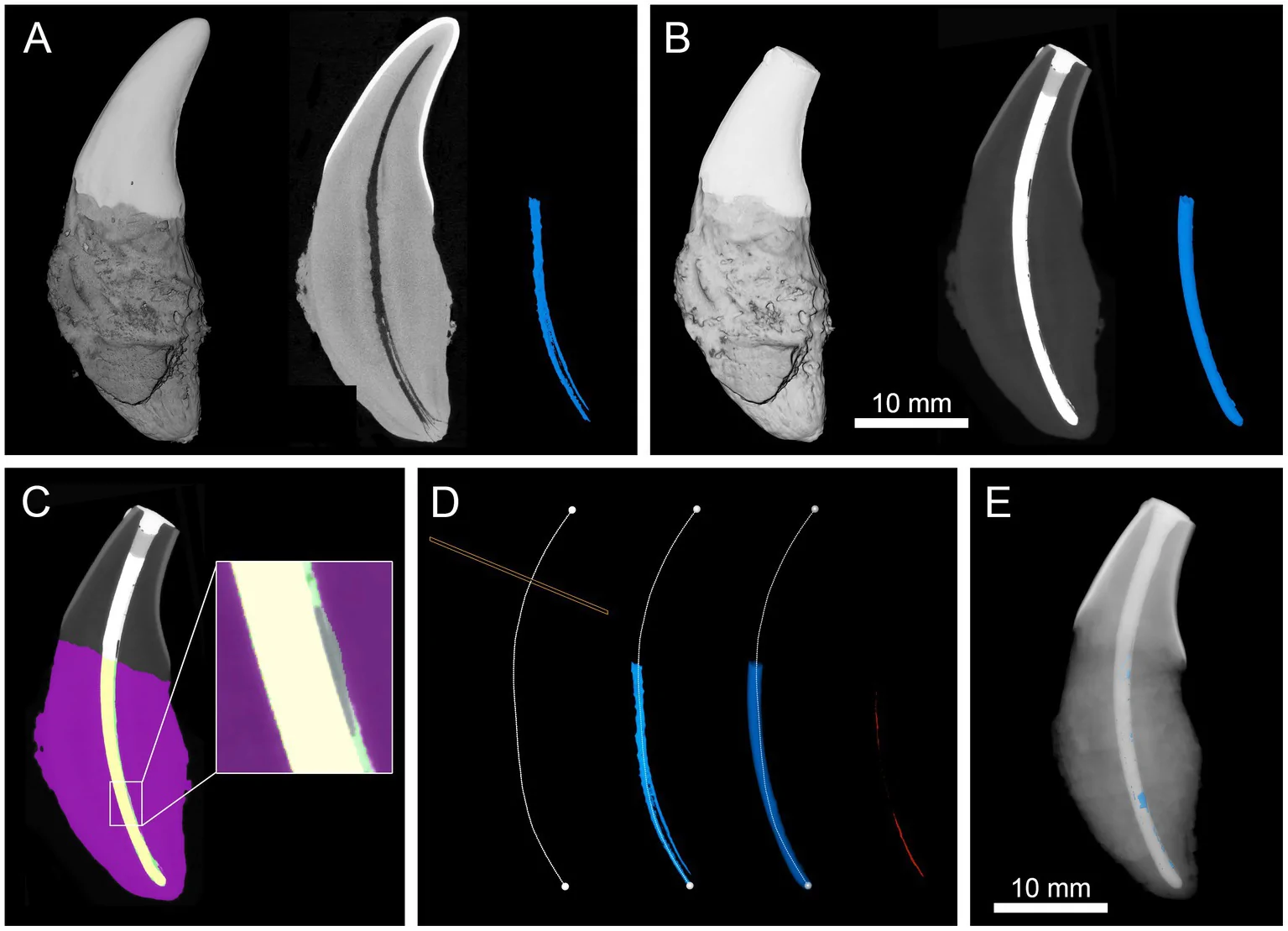
Micro-CT image-processing and analysis workflow for pre- and post-treatment assessment of the root canal system. **(A)** Pre-treatment dataset showing a three-dimensional rendering of the tooth, a longitudinal micro-CT section, and the segmented pre-treatment pulp cavity (blue). **(B)** Post-treatment dataset showing a three-dimensional rendering of the tooth, a longitudinal micro-CT section of the obturated root canal, and the segmented post-treatment pulp cavity (blue). **(C)** Segmentation of the post-treatment dataset using predefined masking thresholds, with tooth structure shown in purple, gutta-percha in yellow, GuttaFlow Bioseal in green, and air in gray; the inset shows a magnified view of the dentin–filling material interface. **(D)** Spatial graph-based analysis of the root canal trajectory, showing from left to right: The B-spline defining the canal centerline and the orientation of a representative perpendicular cross-sectional plane; the three-dimensional pre-treatment pulp segmentation aligned with the spatial graph; the corresponding post-treatment pulp segmentation; and the remaining untreated portion of the pulp cavity (red). **(E)** Post-treatment micro-CT rendering showing segmented internal air voids (blue) within the obturated root canal system. Scale bars = 10 mm.

#### Post-treatment segmentation and material classification

Post-treatment micro-CT datasets were filtered using a non-local means algorithm (interpretation: 3D; spatial SD = 5; intensity SD = 0.2; search window = 10 px; local neighborhood = 3 px). The tooth and the filled pulp were segmented again using the *Amira Segmentation Editor*. Fixed thresholds were used for the segmentation of the tooth (lower threshold: 2000, upper threshold 11,000) and the pulp filling material and gutta percha point (lower threshold: 11001), which showed much higher X-ray attenuation compared to the tooth. Air cavities in the treated pulp were added to the pulp label using manual segmentation tools, thus the segmentation of the pulp represented the entire treated pulp canal. The segmented tooth and pulp were smoothed using the Smooth Labels command (size: 6, mode = 3D volume). Subsequently, the pulp compartment was duplicated to enable segmentation of restorative materials. Separate material classes were defined for gutta-percha point, GuttaFlow bioseal cement and air. Each material was segmented using fixed masking thresholds (air: 0–10,999, cement: 11000–16,000, gutta-percha: 28000–32,767) and region-growing tools, followed by 3D smoothing (*Smooth Labels*, size = 3, mode = 3D volume). Finally, the segmentation of restorative materials was verified by careful visual inspection. The resulting label field was stored for quantitative analysis.

#### Volumetric and surface analysis

To restrict the quantitative analysis to the root portion, the crown of the tooth (region of interest (ROI)) was manually outlined using the tool *Volume edit* and excluded from all label fields (pre-treatment pulp, post-treatment pulp, pulp restoration materials) in the *Segmentation Editor*. Root volumes of the pre- and post-treatment pulp were then analyzed using the *Material Statistics tool*. The *Arithmetic* tool (pre-treatment pulp volume minus treated pulp volume) was used to compute the proportion of pulp that remained untreated. Volume measurements (in mm^3^) were exported from Amira to Microsoft Excel.

The volume of pulp restoration materials was also measured using the *Material Statistics* tool. In addition, a polygon surface model was generated from the restoration material segmentation using the *Generate Surface* tool (algorithm mode: repeatable, smoothing: existing weights). Subsequently, the tool *Surface Area Volume* (mode: patches) was used to calculate interfaces (in mm^2^) between *fill material (GuttaFlow bioseal)/tooth*, *gutta-percha/tooth*, and *air/tooth*.

#### Post-treatment cross-sectional analysis

For cross-sectional evaluation of the treated pulp, the spatial graph from the untreated pulp and the Amira Tcl script used before for the untreated pulp was executed to automatically extract 201 cross-sectional images per sample. These images were again processed using a custom FIJI (ImageJ) scripts to quantify geometric parameters of the pulp and filling interfaces. The resulting data were compiled and analyzed in Microsoft Excel for volumetric and surface comparisons between pre- and post-treatment conditions.

#### File-to-canal diameter analysis

To compare the dimensions of the instrumented root canal with those expected from the final files featuring a 2% taper, the curved pulp length was standardized according to a predefined spline consisting of 201 points. For the file-to-canal diameter analysis, Feret’s diameter obtained from the Fiji analysis was used as the measure of the actual post-treatment canal diameter and was converted from pixels to millimeters according to the respective pixel size. At each of the 201 standardized positions, the Feret’s diameter of the post-treatment canal was compared with the calculated diameter of the final 2%-tapered instrument at the corresponding position, and the difference between both measurements was determined. For each tooth, the mean and median discrepancies across all 201 positions were subsequently calculated and used for statistical comparison between instrumentation groups.

### Statistical analysis

Statistical analyses were performed using IBM SPSS Statistics (Version 29; IBM Corp., Armonk, NY, USA). Continuous variables were summarized descriptively as mean ± standard deviation (SD) and 95% confidence intervals (95% CI), where appropriate.

To evaluate the effects of instrumentation technique on canal geometry, obturation quality and interface characteristics, different statistical models were applied depending on the outcome variable. Differences between canal diameter and instrument size as well as internal air volume were analyzed using linear models including the fixed effects of group (A, B, C), jaw (maxillary vs. mandibular canine teeth), and their interaction (group × jaw). In contrast, the post-treatment/pre-treatment canal volume ratio and surface-based interface measurements were analyzed using one-way analysis of variance (ANOVA), as jaw was not included in these models. No random-effects structure was included in the fitted linear models. Surface analyses comprised quantitative measurements of the interfaces between filling material and tooth structure, gutta-percha, and tooth structure, as well as air and tooth structure. For variables demonstrating non-normal distribution patterns or heteroscedasticity, logarithmic transformation was additionally applied prior to analysis. Estimated marginal means derived from the fitted models were used for pairwise group comparisons. Whenever significant main effects of group were detected, post-hoc comparisons were performed using Sidak’s alpha correction procedure. Additional subgroup analyses according to tooth position (Triadan 104/204 vs. 304/404) were conducted where indicated.

To assess potential operator-dependent effects, independent-samples t-tests were performed comparing volumetric and surface-based outcome variables between the two operators.

Model assumptions were evaluated by visual inspection of residual and Q–Q plots to assess approximate normality and homogeneity of residual variance. All statistical tests were two-sided and statistical significance was defined as *p* < 0.05.

## Results

### File-to-canal diameter discrepancy

The discrepancy between the post-treatment canal diameter and the corresponding final instrument diameter was evaluated using the mean and median differences calculated across the 201 standardized positions for each tooth. Larger values therefore represent a greater discrepancy between the prepared canal diameter and the diameter of the final instrument.

Analysis of the mean discrepancy revealed a significant main effect of group (*F*(2, 24) = 5.94, *p* = 0.008), whereas neither the effect of jaw (*p* = 0.129) nor the group × jaw interaction (*p* = 0.766) reached statistical significance.

Descriptive analysis showed mean discrepancies of 0.162 mm (SD = 0.144; 95% CI: 0.034–0.233) for group A, 0.098 mm (SD = 0.117; 95% CI: 0.000–0.186) for group B, and 0.299 mm (SD = 0.155; 95% CI: 0.208–0.390) for group C, with the highest mean discrepancy observed in group C.

Sidak-adjusted pairwise comparisons demonstrated a significant difference between groups C and B (*p* = 0.010). In contrast, the comparisons between groups C and A (*p* = 0.055) and between groups A and B (*p* = 0.906) did not reach statistical significance.

Analysis of the median discrepancy likewise demonstrated a significant effect of group (*F*(2, 24) = 5.85, *p* = 0.009) and a significant effect of jaw (*F*(1, 24) = 5.09, *p* = 0.033). The interaction between group and jaw was not statistically significant (*p* = 0.729).

Estimated marginal means for the median discrepancy were 0.129 mm (95% CI: 0.043–0.214) for group A, 0.064 mm (95% CI: −0.015–0.144) for group B, and 0.247 mm (95% CI: 0.169–0.325) for group C, again with the largest values observed in group C.

Sidak-adjusted post-hoc comparisons demonstrated a significant difference between groups C and B (*p* = 0.008), whereas the comparisons between groups C and A (*p* = 0.130) and between groups A and B (*p* = 0.609) were not statistically significant.

Subgroup analysis according to jaw revealed a significant effect of group for maxillary canine teeth (104/204; *F*(2, 24) = 3.59, *p* = 0.043), whereas no significant group effect was detected for mandibular canine teeth (304/404; *p* = 0.085).

Overall, both the mean and median analyses consistently showed the greatest discrepancy between the actual post-treatment canal diameter and the corresponding final instrument diameter in group C. Significant pairwise differences were identified only between groups C and B, whereas comparisons involving group A were not statistically significant after Sidak correction.

### Incidence of procedural complications

Procedural complications occurred in 8 of 30 teeth (26.7%), accounting for a total of 10 individual incidents. In several cases, more than one type of complication was recorded for the same tooth.

The most frequent complications were associated with the use of Gates-Glidden burs (4 cases, 40%), followed by ISO size 15 files (3 cases, 30%), Path Finder instruments (2 cases, 20%) and ISO size 70 files (1 case, 10%).

Fragment removal was successful in 5 of the affected 8 teeth (62.5%), whereas in 3 cases (37.5%), retrieval was unsuccessful and a replacement tooth had to be used for further analysis.

When stratified by operator experience, 7 of the 10 complications (70%) occurred in procedures performed by a pre doctoral specialist (operator no.1), while 3 incidents (30%) occurred during instrumentation by a board-certified veterinary dentist DAVDC/DEVDC (operator no.2).

### Volumetric analysis

The post-to-pre canal volume ratio, calculated as the ratio of the post-treatment canal volume to the pre-treatment canal volume, did not differ significantly among the three instrumentation techniques (*F*(2,27) = 0.79, *p* = 0.464). Mean post-to-pre canal volume ratios were 11.47 ± 4.91 in group A, 11.33 ± 8.29 in group B, and 8.40 ± 4.60 in group C. Pairwise comparisons using Sidak-adjusted post-hoc tests revealed no significant differences between any of the groups (A vs. B: *p* = 1.000; A vs. C: *p* = 0.620; B vs. C: *p* = 0.652; Figure 5).

**Figure 5:**
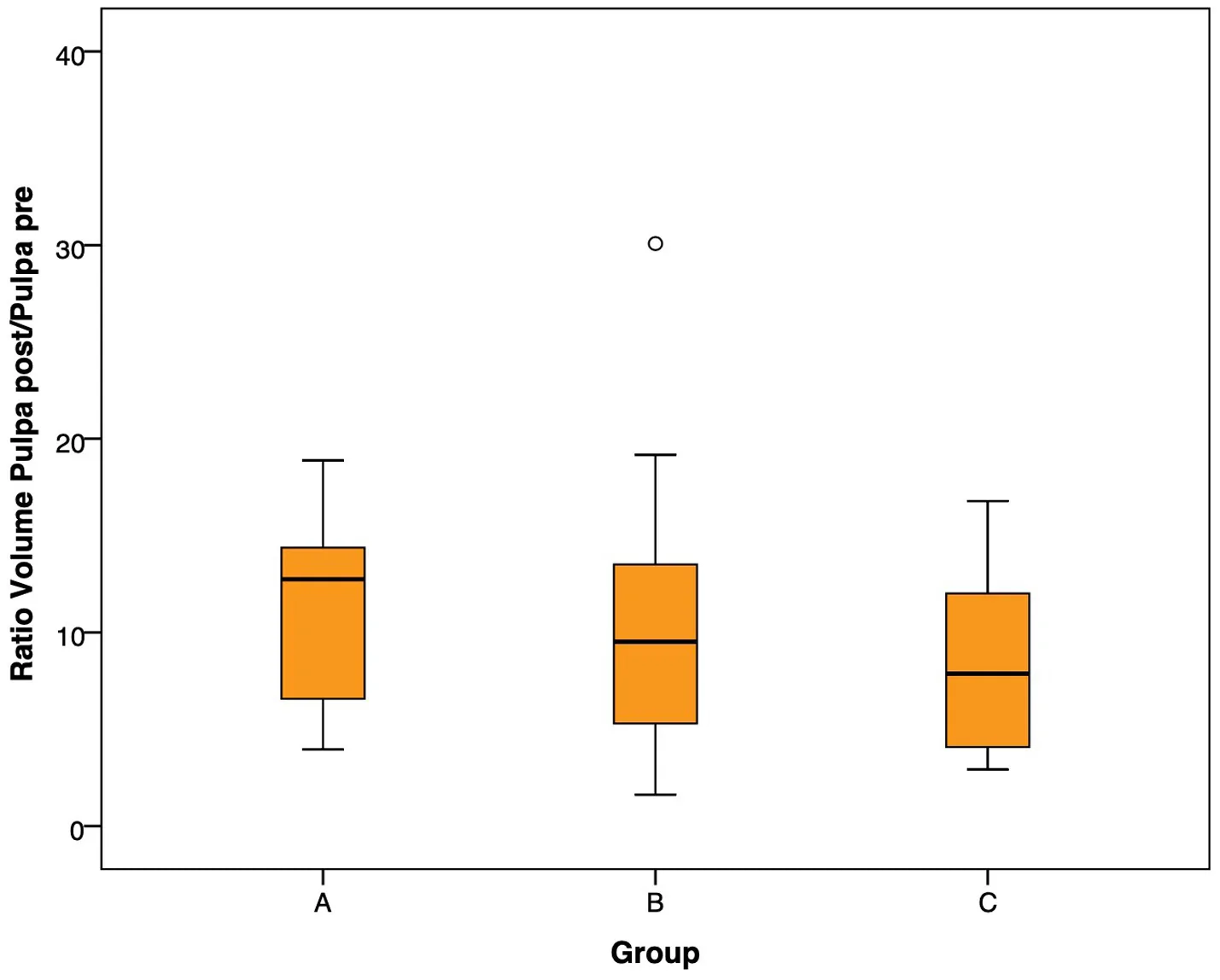
Post-to-pre root canal volume ratio according to instrumentation group. Group A: Control cut rotary flex Ni-Ti. Group B: Vet flex & preserve Ni-Ti. Group C: Manual Ni-Ti hand instrumentation.

### Air volume analysis

A significant effect of group was observed for total air volume (*F*(2, 24) = 4.83, *p* = 0.017).

Pairwise Sidak-adjusted comparisons revealed no significant difference between groups C and A (*p* = 0.060), whereas group C exhibited significantly higher air volumes than group B (*p* = 0.028). No significant difference was observed between groups A and B (*p* = 0.994).

Neither jaw (*p* = 0.966) nor the group × jaw interaction (*p* = 0.140) was significant. Estimated marginal means were 0.61 mm^3^ for group A, 0.46 mm^3^ for group B and 2.15 mm^3^ for group C. Thus, group C demonstrated the highest volume of internal voids or unfilled canal spaces, indicating a less homogeneous obturation pattern compared with groups A and B (Figure 6).

**Figure 6:**
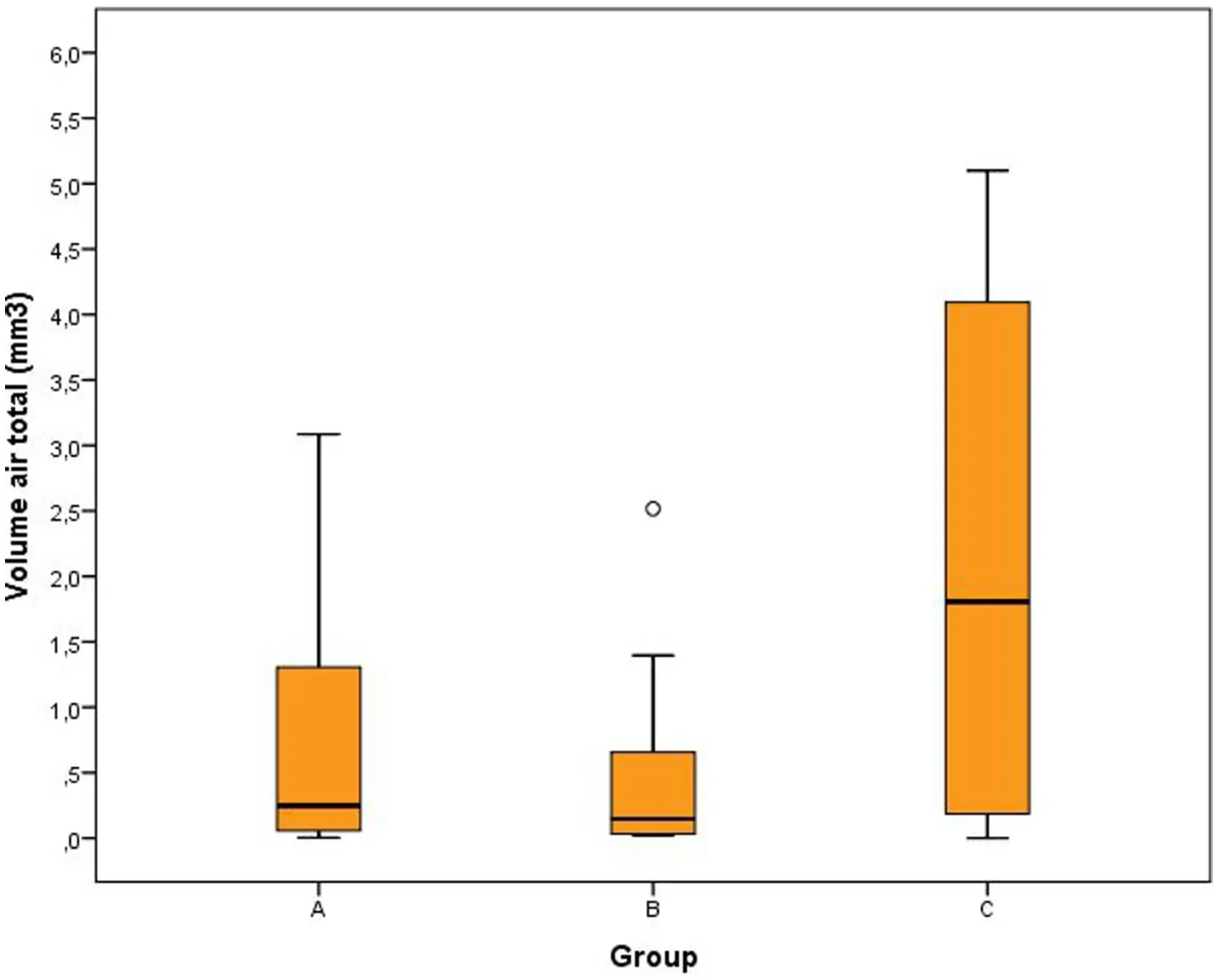
Internal air volume within the obturated root canal system according to instrumentation group (mm^3^). Group A: Control Cut Rotary Flex Ni-Ti. Group B: Vet Flex & Preserve Ni-Ti. Group C: Manual Ni-Ti hand instrumentation.

### CBCT and micro-CT comparison

CBCT imaging revealed radiolucent areas at the dentin-filling material interface in multiple specimens, which could be interpreted as potential voids or interfacial defects. However, comparative analysis using high-resolution micro-CT demonstrated no corresponding voids, debris accumulations, or air inclusions at these locations. Instead, the micro-CT datasets showed continuous adaptation of the obturation material to the canal walls, indicating that these radiolucent zones did not represent true structural deficiencies.

Quantitative micro-CT analysis further confirmed that internal air volumes were limited and predominantly localized within the canal system rather than at the dentin-filling interface. The absence of structural discontinuities at sites corresponding to CBCT radiolucencies suggests that these findings are attributable to imaging-related contrast effects rather than incomplete obturation or residual debris.

### Surface analysis

Surface analyses showed no significant differences among groups for any of the evaluated parameters: filling material surface/tooth (*F*(2, 27) = 0.31, *p* = 0.734), gutta-percha surface/tooth (*F*(2, 27) = 0.20, *p* = 0.818) and air surface/tooth (*F*(2, 27) = 0.16, *p* = 0.855).

These findings indicate that surface characteristics of the obturation materials and interfaces were comparable across all groups, despite volumetric differences in internal air volume.

### Operator comparison

Independent-samples t-tests comparing operator performance showed no significant differences in volumetric parameters (*p* > 0.25 for all). However, the surface area of the filling material per tooth differed significantly between operators (t(28) = 2.18, *p* = 0.038), with operator no.1 (JW) producing slightly larger filling surfaces than operator no. 2 (MES). This finding suggests minor operator-dependent variability in obturation or segmentation, while overall volumetric outcomes remained consistent.

Taken together, the volumetric analyses revealed no significant group differences in total canal volume after treatment but group C showed a consistent tendency toward increased air volume within the obturated canals. Surface-based metrics did not vary significantly among groups and inter-operator variability was minimal.

## Discussion

The present study evaluated different endodontic instrumentation techniques in canine teeth using high-resolution micro-CT and assessed their effects on canal geometry, obturation quality and procedural safety. The findings provide insight not only into shaping characteristics but also into the interpretation of postoperative imaging.

With regard to the CBCT assessment, the results support the assumption that radiolucent areas observed at the dentin-filling material interface predominantly represent imaging-related artifacts rather than true treatment deficiencies. Comparative analysis using high-resolution micro-CT demonstrated that these radiolucent zones corresponded to contrast transitions between materials of differing radiodensity and did not reflect residual necrotic dentin, persistent debris or entrapped air within the canal system (21). The absence of corresponding voids, debris accumulations or structural discontinuities in the micro-CT datasets strongly suggests that the CBCT findings were attributable to beam-hardening effects or contrast-related imaging phenomena rather than inadequate obturation or insufficient debridement. These observations emphasize the need for cautious interpretation of CBCT findings in veterinary endodontic follow-up and highlight the added diagnostic value of high-resolution imaging for distinguishing true structural defects from modality-related artifacts (22) (Figures 7A,B).

**Figure 7:**
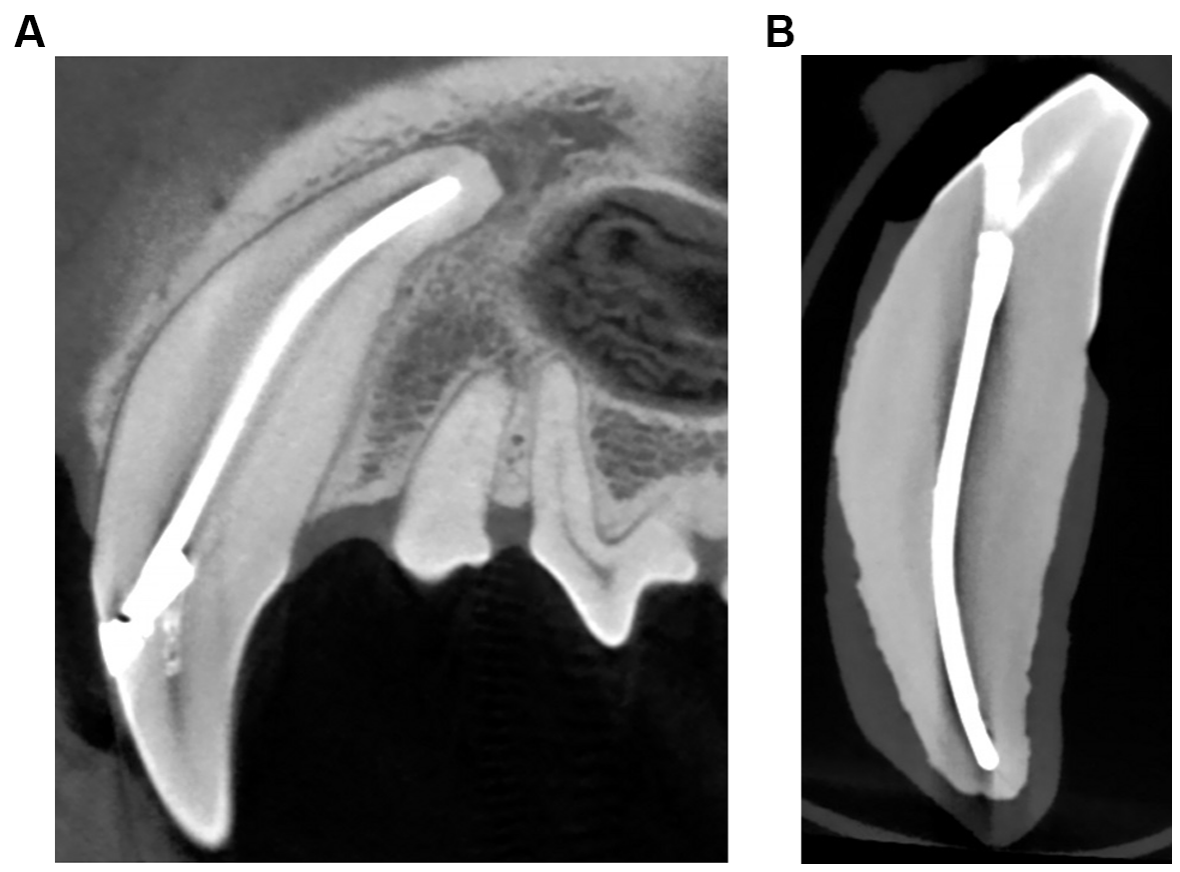
Representative postoperative CBCT images demonstrating similar radiolucent zones at the dentin–filling material interface under clinical and experimental conditions. **(A)** CBCT image of an endodontically treated canine tooth from a clinical patient showing a radiolucent zone at the dentin–filling material interface. **(B)** CBCT image of specimen C03 from the present *in vitro* study showing a comparable radiolucent appearance at the dentin–filling material interface.

Compared with conventional intraoral radiography, CBCT inherently provides three-dimensional visualization of root canal anatomy and avoids limitations related to superimposition and projection inherent to two-dimensional imaging. Previous studies have demonstrated the diagnostic value of CBCT for the assessment of root canal morphology and endodontic treatment outcomes (23, 24). However, the present study did not systematically compare CBCT with conventional intraoral radiography. Rather, our findings demonstrate that radiolucent areas observed at the dentin-filling material interface on CBCT require careful interpretation, as corresponding high-resolution micro-CT evaluation indicated that not all apparent radiolucencies represented true structural discontinuities. Awareness of such imaging-related phenomena is therefore important when interpreting postoperative CBCT examinations.

At our institution, CBCT examinations are routinely performed before and after root canal treatment and are complemented by intraoperative intraoral radiographs for continuous procedural documentation. Follow-up examinations include both imaging modalities to facilitate assessment of treatment success, failure, or the absence of radiographic evidence of disease. This combined imaging approach may enhance diagnostic confidence, particularly in complex cases where two-dimensional imaging alone may be insufficient (23, 25).

Beyond diagnostic assessment, the present findings provide insight into the biomechanical consequences of different instrumentation strategies for root canal preparation. High-resolution three-dimensional imaging enabled a detailed assessment of canal enlargement and dentin removal patterns, allowing a more comprehensive evaluation of shaping quality and preservation of radicular dentin (26). Maintaining an appropriate balance between effective canal enlargement for adequate debridement and preservation of dentin is considered essential for the long-term structural integrity of endodontically treated teeth (27).

Machine-driven Ni-Ti systems tended to produce a smaller discrepancy between file size and actual canal diameter than manual instrumentation. Following Sidak correction, however, a statistically significant pairwise difference was observed only between groups B and C (28). Although no significant differences in the post-to-pre canal volume ratio were observed among the three instrumentation techniques, manual instrumentation tended to result in higher internal air volumes. Pairwise comparisons demonstrated significantly greater air volumes in group C than in group B, whereas the difference between groups C and A did not remain statistically significant after Sidak adjustment. The absence of statistical significance between groups A and C after correction for multiple comparisons suggests that, although a similar trend was observed, the magnitude of the difference was insufficient to demonstrate a robust effect under the more conservative Sidak procedure.

These findings suggest that the advantages of mechanical instrumentation are not primarily attributable to greater overall canal enlargement but rather to more controlled and reproducible shaping.

Improved canal centering and a closer correspondence between instrument size and actual canal dimensions may facilitate more effective debridement while minimizing unnecessary removal of radicular dentin (4, 26). Consequently, preparation quality appears to depend less on the extent of canal enlargement than on the precision with which the original canal anatomy is respected (23). Collectively, these findings indicate that shaping precision, rather than the extent of canal enlargement, may represent a key determinant of preparation quality in veterinary endodontics.

While the present study primarily focused on volumetric and geometric changes following instrumentation, volumetric enlargement does not necessarily reflect the proportion of the canal wall mechanically contacted by the instruments. Previous micro-CT studies in human endodontics have demonstrated that areas of the root canal wall may remain untouched despite substantial canal enlargement (26). Therefore, volumetric changes and instrumented canal wall surface should be considered complementary measures of shaping efficacy.

An additional consideration is the difference in access design between the instrumentation protocols. An auxiliary mesial access was used in group C but not in groups A and B. Despite the flexibility and shape-memory properties of Ni-Ti instruments, an auxiliary access is recommended for the instruments used in group C to provide a more direct approach to the apical portion of the curved canal. Appropriate access design is an important component of endodontic treatment in canine teeth, with straight-line access toward the apex facilitating instrument insertion while minimizing unnecessary removal of tooth structure (29). Mesial access has also been described as a standard approach for endodontic treatment of canine teeth in dogs (10). Therefore, the additional access in group C would theoretically be expected to facilitate rather than impair instrumentation. Nevertheless, because access design was not standardized across all groups, its potential influence on shaping outcomes cannot be excluded. The observed differences should therefore be interpreted as reflecting the respective instrumentation protocols as a whole rather than the instrument systems alone.

In human endodontics, machine-driven Ni-Ti instrumentation has demonstrated advantages in shaping ability, procedural safety, and preparation efficiency compared with manual instrumentation (4, 28). However, an important distinction must be made between the continuous rotary motion evaluated in many human endodontic studies and the reciprocating motion used for the machine-driven systems in groups A and B of the present study. Reciprocating kinematics may increase the cyclic fatigue resistance of Ni-Ti instruments compared with continuous rotation, which may be particularly relevant during instrumentation of long and curved root canals (13). Accordingly, findings from studies evaluating continuously rotating Ni-Ti systems should not be directly extrapolated to the reciprocating systems investigated in the present study.

In veterinary endodontics, the long and frequently curved root canals of canine teeth present particular challenges for mechanical preparation. In this anatomical setting, the greater flexibility of Ni-Ti compared with stainless-steel instruments may be advantageous, as it facilitates maintenance of the original canal trajectory and reduces the tendency for canal transportation in curved canals (30). These anatomical and mechanical considerations are particularly relevant when interpreting differences between instrumentation techniques in canine teeth (2, 6).

Nevertheless, the mechanical properties of Ni-Ti hand instruments also impose inherent limitations. Their superelasticity and shape-memory behavior, while facilitating adaptation to canal curvature, may limit the ability to deliberately direct the instrument against specific canal walls and may therefore result in incomplete mechanical contact with portions of the canal surface. This characteristic may have contributed to the less standardized shaping and greater internal air volumes observed in group C, although canal-wall contact was not directly quantified in the present study. Accordingly, the findings for group C should be interpreted specifically in the context of manual Ni-Ti instrumentation and should not be generalized to manual instrumentation using pre-curved stainless-steel files.

The near-significant interaction between instrumentation group and jaw may also reflect underlying anatomical variability in root morphology. Previous three-dimensional micro-CT analyses of canine teeth have demonstrated considerable interindividual variation in root canal curvature, with curvature patterns influenced by body size and individual root morphology (30). Even in the absence of consistent volumetric differences between maxillary and mandibular canines, subtle variations in canal trajectory and curvature may alter the mechanical behavior of instruments during preparation. Increased curvature or complex canal pathways can render shaping more technique-sensitive, particularly when using manual instrumentation that is more prone to deviation and transportation (31). These anatomical characteristics may increase the technical difficulty of canal preparation and predispose to procedural errors, particularly during manual instrumentation (30). Such anatomical variability may partly explain the observed trend toward jaw-dependent differences in volumetric response.

The increased internal air volume observed in group C most likely reflects less homogeneous obturation, which is commonly associated with less standardized canal geometry (32–34). Although all groups underwent identical obturation procedures, the more reproducible canal geometry achieved with machine-driven instrumentation may have facilitated improved adaptation of the filling material and consequently reduced the occurrence of internal voids (18).

Only limited quantitative evidence is currently available comparing different endodontic instrumentation techniques in canine teeth using high-resolution micro-CT. Previous studies have primarily focused on the anatomical variability of canine root canal systems and have demonstrated substantial variation in canal morphology that may complicate complete debridement and obturation (1, 2). The present study expands upon these observations by providing quantitative high-resolution micro-CT evidence that machine-driven instrumentation achieves more standardized canal preparation and fewer internal voids than manual instrumentation.

### Technical and clinical implications

The observed differences in air volume and canal-file discrepancy have practical clinical relevance. Air voids and unfilled canal spaces may compromise the hermetic seal and increase the risk of bacterial leakage, which remains a key determinant of long-term endodontic success (23, 35). The results therefore support the clinical use for machine-driven Ni-Ti systems in veterinary endodontics, particularly for strategic teeth such as canines and carnassials.

Moreover, the study indicates that operator experience may play an important role in the occurrence of procedural errors, as most complications were observed during treatments performed by a less experienced operator. Although the sample size was limited, instrumentation-related complications occurred in approximately one quarter of the specimens, with some teeth exhibiting multiple procedural failures. These findings emphasize the technical challenges associated with endodontic preparation of canine teeth and suggest that operator skill level may substantially influence the incidence of instrument separation and related complications.

Beyond the quantitatively analyzed outcomes, several qualitative observations were noted during handling of the instruments and are reported here as incidental, hypothesis-generating findings rather than as a systematic between-group comparison. In addition to complete instrument separation, a distinct pattern of plastic deformation was occasionally observed, characterized by visible unwinding of the helical flutes and elongation of the active working segment prior to fracture. This pattern is consistent with torsional stress accumulation, which occurs when the file tip binds within the canal while the coronal portion continues to rotate, and may act as a macroscopic warning sign preceding instrument failure. Furthermore, visual inspection of newly unpacked instruments occasionally revealed fine metallic debris adherent to the cutting flutes prior to use; if not removed, such particles could conceivably be introduced into the canal as foreign material. As these observations were not standardized or quantified, they should be interpreted with caution; nevertheless, early recognition of flute unwinding and careful inspection of new instruments may represent simple preventive measures, and their systematic evaluation would be a worthwhile objective for future studies (4, 36) (Figures 8A–C).

**Figure 8:**
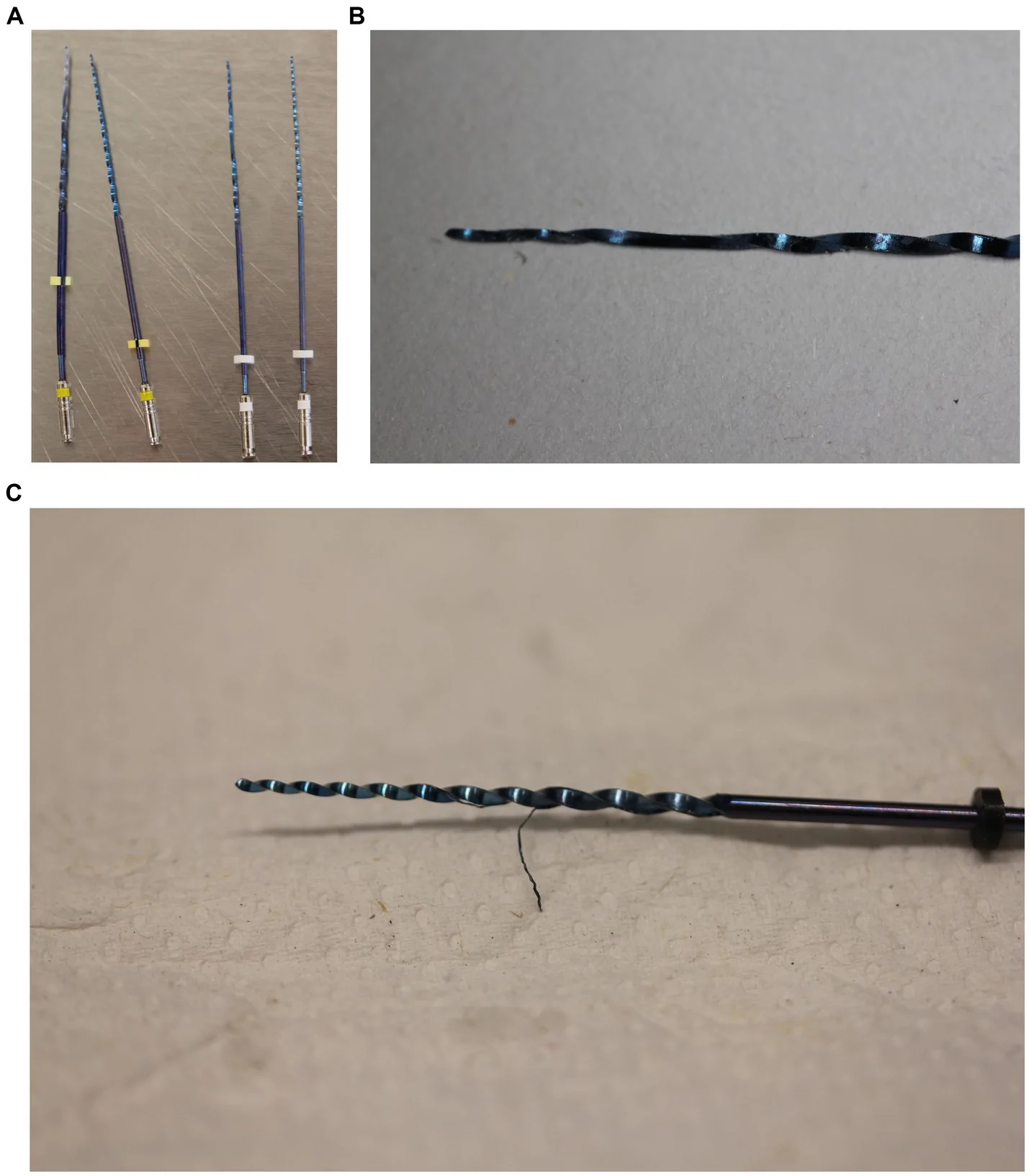
Representative qualitative observations of endodontic instruments during visual inspection. **(A)** Plastic deformation characterized by unwinding of the helical flutes and elongation of the active working portion. **(B)** Close-up view of the deformed instrument showing flute unwinding. **(C)** Fine metallic debris adherent to the cutting flutes of a newly unpacked instrument prior to use.

Collectively, these observations highlight the importance of both operator proficiency and preparation quality for achieving predictable endodontic outcomes (37). This interpretation is consistent with human studies demonstrating a strong correlation between operator training, technical performance and endodontic success (10, 38, 39).

However, the presence of significantly greater internal void volume in group C may indicate incomplete adaptation of the obturation material, potentially resulting from the less standardized canal geometry achieved by manual instrumentation, even though the same Ni-Ti alloy was used for both hand and motor-driven files.

### Limitations and future directions

The present study has several limitations that should be considered when interpreting the findings. The study used extracted canine teeth, which allowed standardized preparation and imaging but did not account for *in vivo* conditions such as periodontal ligament support, pulp vitality and physiological moisture content.

An additional consideration concerns the hydration state of the specimens during imaging. Pre-treatment scans were acquired with the teeth immersed in 70% ethanol, whereas after embedding, access preparation, instrumentation, obturation, and restoration, the post-treatment scans were performed without re-immersion. A degree of dehydration of the dental hard tissues between the two scans can therefore not be entirely excluded, and drying of root dentin has been shown to induce dentinal microcracks that may, in principle, affect canal measurements (40). However, no dentinal microcracks were observed in the high-resolution post-treatment micro-CT datasets, suggesting that dehydration was unlikely to have caused substantial structural damage detectable at the spatial resolution used in the present study. In addition, all specimens underwent an identical handling and imaging sequence; therefore, any residual effect would be expected to apply similarly across groups and is unlikely to have systematically biased between-group comparisons. The obturated and coronally restored canals may also have limited drying of the internal canal surface. Nevertheless, absolute canal dimensions should be interpreted with appropriate caution, and future studies maintaining a consistent hydration state during pre- and post-treatment imaging would help to further minimize this potential source of bias.

Furthermore, the relatively small sample size, with 10 teeth per instrumentation group, represents an important limitation. As the sample size was constrained by the availability of corresponding pre-treatment micro-CT datasets rather than determined by an *a priori* power analysis, the study may have been underpowered to detect smaller differences between instrumentation techniques. Accordingly, non-significant findings should not necessarily be interpreted as evidence of equivalence between techniques. Interindividual anatomical variability may have further contributed to variability within the relatively small groups. Future studies should include larger sample sizes with prospective sample-size calculations and clinical *in vivo* investigations using CBCT to further evaluate the clinical translatability of the present findings. Additionally, longitudinal studies evaluating sealing ability and fracture resistance after different instrumentation methods would complement the present morphological analysis.

Material segmentation based on fixed gray-level thresholds may have introduced minor inaccuracies in air volume quantification, despite careful validation, at the voxel level by the study operator.

Nevertheless, the standardized imaging and analysis protocol applied to all specimens is likely to have minimized this potential source of error. Finally, although the operator factor showed limited influence on volumetric data, the observed trend toward more procedural complications by less experienced operators emphasizes the importance of specialized endodontic training in veterinary dentistry.

## Conclusion

Within the limitations of this *in vitro* study, machine-driven Ni-Ti systems provided more reproducible root canal preparation and more homogeneous obturation than manual Ni-Ti hand files in canine teeth. First, although the post-to-pre canal volume ratio did not differ significantly among instrumentation techniques, manual instrumentation was associated with greater discrepancies between instrument size and canal geometry, indicating less standardized canal preparation. Second, the increased internal air volume observed with manual instrumentation suggests less homogeneous obturation compared with the machine-driven techniques. Third, radiolucent interface zones observed on CBCT predominantly represented imaging-related phenomena rather than true structural deficiencies when assessed using high-resolution micro-CT, emphasizing the need for cautious interpretation of postoperative CBCT findings. Collectively, these findings support the use of machine-driven instrumentation for more predictable root canal preparation while highlighting the diagnostic limitations of CBCT in assessing the dentin–filling material interface. Further studies are warranted to investigate the long-term biological and mechanical outcomes of different instrumentation techniques under clinical conditions.

## Data Availability

No restrictions apply to the availability of the dataset beyond practical constraints related to file size. The raw micro-CT and CBCT image volumes are large and are therefore not deposited in a public repository, but the full datasets analyzed in this study are available from the corresponding author upon reasonable request. Requests to access the datasets should be directed to johanna.wunderl@gmx.net.
