# Supplementary material for: Machine-driven vs. manual nickel-titanium instrumentation of canine teeth in dogs: a high resolution micro-CT evaluation of canal geometry and obturation quality
Source: Front Vet Sci. 2026 Sep 14;13:1960227. doi: 10.3389/fvets.2026.1960227 (PMC13616723; doi:10.3389/fvets.2026.1960227)
Supplement: Supplementary file 1 [file Table_1.DOCX]

|  | **Material / Instrument** | **Manufacturer** | **City, Country** | **Specifications / Notes** |
| --- | --- | --- | --- | --- |
| a | Control Cut Rotary Flex NiTi files | iM3 | Irland | machine-driven instrumentation (Group A) |
| b | Endomotor C Flex | iM3 | Irland | endomotor |
| c | VET Flex&Preserve NiTi rotary files | Dentsply Sirona | USA | machine-driven instrumentation (Group B) |
| d | NiTi K-Reamer hand files | iM3 | Irland | manual instrumentation (Group C) |
| e | Polycarbonate block | Epodex (Epodex Eco) | Germany | embedding blocks |
| f | Round diamond bur #2 (1 mm) | Komment Dental | Germany | access cavity |
| g | Gates-Glidden burs (Size 1–6) | iM3 | Irland | coronal enlargement |
| h | Glyde File Prep | Dentsply Sirona | USA | canal lubricant/conditioner |
| i | Barbed broach (Size 1–5) | iM3 | Irland | pulp extirpation |
| j | Side-vented irrigation needle 27G, 60 mm | iM3 | Irland | irrigation |
| k | Sodium hypochlorite 5.25% | Speiko | Germany | irrigant |
| l | Sterile sodium chloride 0.9% | B. Braun | Braun | irrigation |
| m | EDTA 17% | Speiko | Germany | final irrigation, smear layer removal |
| n | Paper points | iM3 | Irland |  |
| o | Gutta-percha points | iM3 | Irland | single-cone obturation |
| p | GuttaFlow bioseal | Coltene/ROEKO | Langenau, Germany | sealer |
| q | Plugger | Hu-Friedy | USA | vetical compation |
| r | Excavators | Hu-Friedy | USA | cavity cleaning |
| s | Glass ionomer cement (Fuji II Capsule) | GC | Germany | restoration |
| t | Adhese Universal VivaPen | Ivoclar | Liechtenstein | bonding agent |
| u | Tetric EvoCeram | Ivoclar | Liechenstein | composite |
| v | Polishing kit (Super-Snap Rainbow technique kit) | Shofu | Germany |  |
| w | G-Coat Plus / Nanoseal | GC Corporation | Germany | surface sealant |
