# Supplementary material for: Machine-driven vs. manual nickel-titanium instrumentation of canine teeth in dogs: a high resolution micro-CT evaluation of canal geometry and obturation quality
Source: Front Vet Sci. 2026 Sep 14;13:1960227. doi: 10.3389/fvets.2026.1960227 (PMC13616723; doi:10.3389/fvets.2026.1960227)
Supplement: Supplementary file 2 [file Table_2.DOCX]

FF	First file

LF	Last file

CBCT	Cone-beam computed tomography

Micro-CT	Micro-computed tomography

DICOM	Digital Imaging and Communications in Medicine

TCL	Tool command language

Ni-Ti file	nickel titanium file

GG	Gates Glidden

EVDC/AVDC	European/American veterinary dental college

ISO	International Organization for Standardization

LSD	Least Significant Difference

SE	Standard Error

SD	Standard Deviation

CI	confidence interval

F	F-Wert

CEJ	cementoenamel junction

NaOCl	sodium hypochlorite

NaCl	sodium chlorite

EDTA	ethylenediaminetetraacetic acid
